# A case report of a young girl with recurrent hematuria: a missed diagnosis - renal nutcracker syndrome

**DOI:** 10.1186/s12882-019-1508-6

**Published:** 2019-09-05

**Authors:** Haifa Ali Bin Dahman, Ali Omer Aljabry

**Affiliations:** 1 Pediatric Department, Hadhramout University College of Medicine, Mukalla, Hadhramout governorate Yemen; 2 Neuropsychiatry clinic, Mukalla, Hadhramout governorate Yemen

**Keywords:** Aortomesenteric angle, Hematuria, Left renal vein, Nutcracker syndrome

## Abstract

**Background:**

Nutcracker syndrome is an easily missed cause of hematuria in children. It is characterized by left renal vein entrapment between the abdominal aorta and the superior mesenteric artery causing renal venous hypertension. Intermittent hematuria and orthostatic proteinuria with or without abdominal or flank pain are the common clinical manifestations. Presence of variable non-specific symptoms and non-significant physical findings results in a delayed diagnosis.

**Case presentation:**

We present a ten -year -old girl with four episodes of painless gross hematuria and recurrent microscopic hematuria since the age of two years. Doppler ultrasound showed left renal vein compression while 3 D computerized tomography angiography confirmed the diagnosis of an anterior nutcracker. The patient was conservatively treated with nutritional support (pediasure complete formula and high calorie food), iron supplements and followed up, monitored for anemia, hypertension and renal insufficiency.

**Conclusion:**

Nutcracker syndrome is a rare cause of recurrent gross hematuria in children. A high index of suspicion and proper imaging is needed to reach a proper diagnosis and avoid the psychological and financial stress on the family.

## Background

Macroscopic hematuria in children is a common cause of referral to hospital [1]. Common causes of hematuria including infection, glomerulonephritis, stones, and hypercalciuria can be easily diagnosed with proper history, physical and laboratory examination. Some rare causes as nutcracker syndrome (NCS) may need an extensive workup [1]. Nutcracker syndrome is characterized by a set of signs and symptoms secondary to compression of the left renal vein (LRV) either in the acute anatomic angle between the abdominal aorta (AA) and the origin of the superior mesenteric artery (SMA) known as anterior NCS [2–5] or in a retro-aortic position between AA and vertebral column, which is named posterior NCS [2, 3, 6–9].

NCS is an easily missed diagnosis because of its variability in presentation ranging from no symptoms to a complex set of symptoms [2]. Variable combinations of symptoms including left flank pain, abdominal pain, hematuria (macroscopic or microscopic) and proteinuria have been reported [3, 6].

Diagnosis of NCS depends on the demonstration of LRV compression using Doppler ultrasound (US), computerized tomography angiography (CTA), magnetic resonance angiography (MRA) and venography [1]. Treatment of NCS is controversial, it includes observation with spontaneous remission for mild cases and different surgical approaches for severe cases [2, 3, 10]. We present a case of ten- year- old Yemeni girl with unexplained recurrent painless gross hematuria diagnosed as anterior NCS. To our knowledge, this is the first reported case in Yemen.

## Case presentation

A ten- year- old girl, presented to our clinic with a history of four episodes of unexplained painless gross hematuria since the age of two years. Each episode had spontaneous gradual resolution, while the period between the episodes was from 3 months to 3 years. She denied any other signs or symptoms including fever, headache, convulsions, periorbital edema, joint pain, abdominal or flank pain, weight loss, skin rash, bleeding tendency, dysuria or intermittent micturition. Hematuria was not preceded by upper respiratory tract infection or related to urinary trauma or exercises. There was no history of hospital admission or blood transfusion. She had a history of allergy to mosquito bites, negative history of deafness in the family and a positive history of renal stones in the family (mother & uncle). Urine analysis (UA), kidney function tests (KFT) and renal ultrasounds were repeatedly normal. She was treated as a case of urinary tract infection by different specialists in outpatient clinics.

On examination she was slightly pale. Her weight and height were 20 kg (below 3rd centile) and 128 cm (below 10th centile) respectively. Her blood pressure (BP) was 100/70 mmHg and systemic examination was normal.

Laboratory investigations showed mild iron deficiency anemia (Hb 10 g/dl), erythrocyte sedimentation rate 50 mm/hr. Other investigations including KFT, calcium, uric acid, C- reactive protein (CRP), sickling test, C3, C4, anti-nuclear antibodies, antistreptolysin O, spot urinary calcium/ creatinine ratio (0.08) and 24 h urine collection for calcium and oxalate, all were normal.

Urine analysis confirmed gross hematuria (RBCs full number), albumin + 1, white blood cells 10-12/ high power field (HPF), negative nitrite, and absent casts. Microscopic hematuria was noticed after resolution of gross hematuria. Screening UA for the patient family members was negative. A plain abdominal x-ray was normal. Renal US showed normal kidney measurements and echogenicity and it excluded hydronephrosis, masses or stones.

**Fig. 1:**
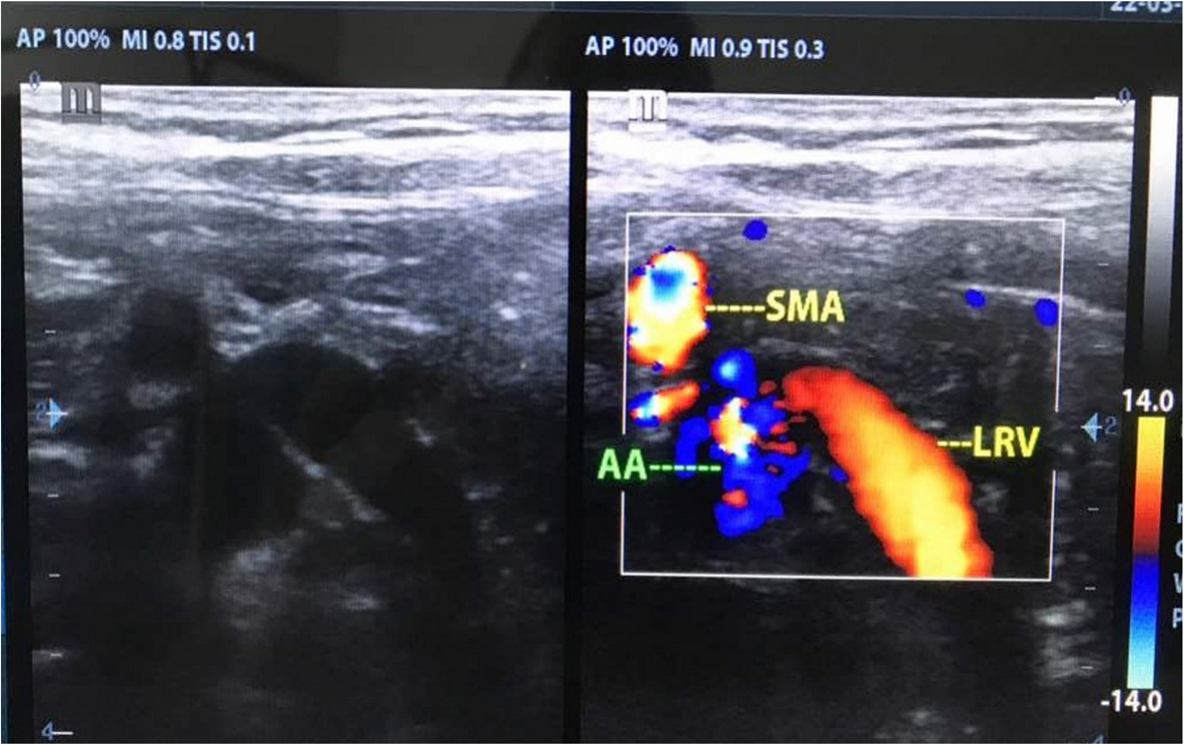
Doppler ultrasound of the left renal vein showed compression of the left renal vein between superior mesenteric artery and abdominal aorta with dilation of the proximal part of the left renal vein

Doppler US of the left renal vein revealed compression of the LRV between the superior mesenteric artery and abdominal aorta with dilation of the proximal part of the LRV (Fig. 1). The diagnosis was confirmed by CTA which showed a reduced aortomesenteric angle of 31.4° (Normal 38–65°) and entrapment of LRV between AA and SMA with dilatation of the proximal part (Towards the inferior vena cava) of LRV (6 mm) in comparison to.

**Fig. 2:**
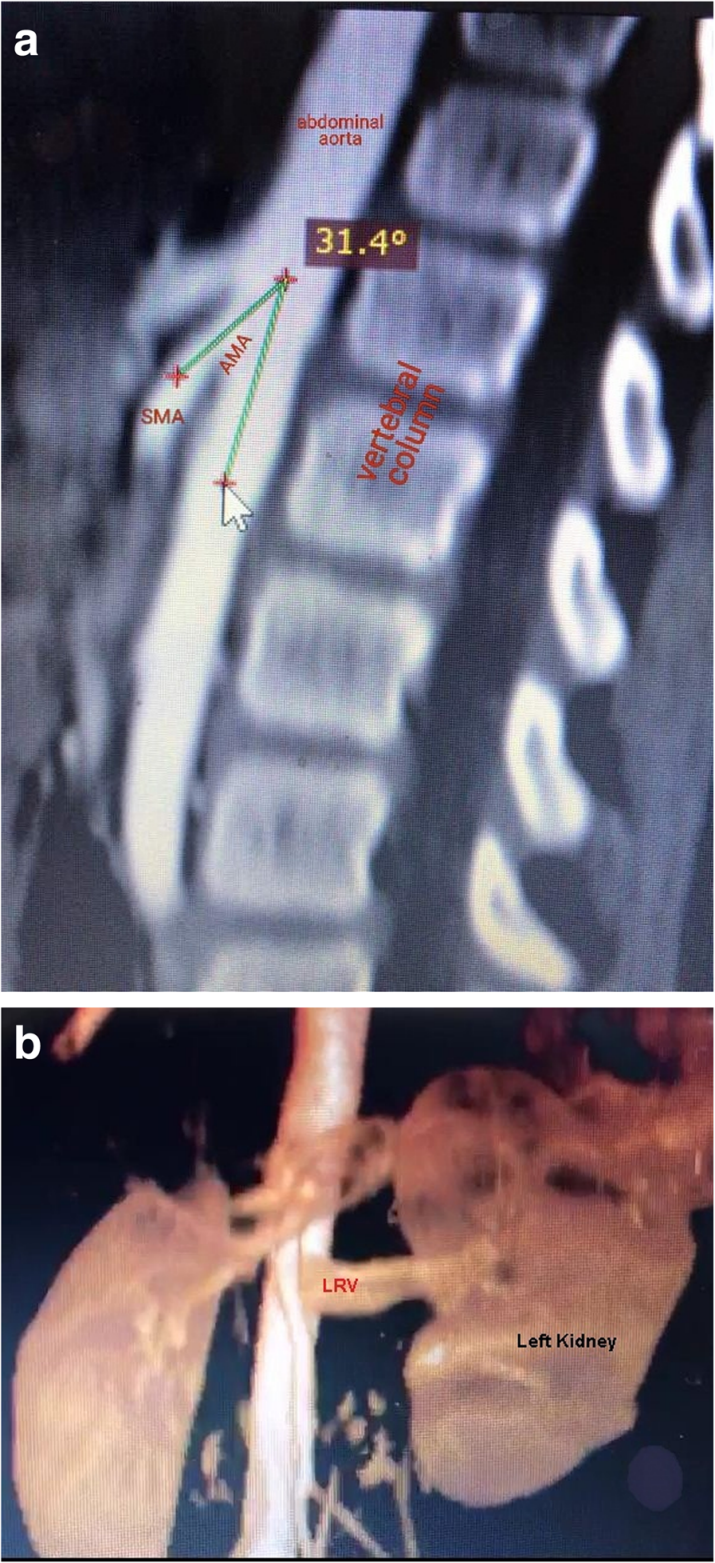
sagittal computerized tomography angiography with 3D reconstruction. Notes: (**a**) Sagittal computerized tomography angiography shows reduced aortomesenteric angle of 31.4° with entrapment of left renal vein between abdominal aorta and superior mesenteric artery. (**b**) 3D reconstruction of the same computerized tomography angiography showed the entrapment of left renal vein with dilatation of the proximal part of left renal vein

< 1 mm distally; towards the hilum of the kidney (Post SMA crossing). No collateral circulations were detected (Fig. 2 a, Fig. 2 b). These anatomical findings in addition to the patients’ clinical presentation, all were consistent with the diagnosis of anterior nutcracker syndrome.

We discussed the case with a nephrologist and a urologist, and later informed the parents about the prognosis of the case. Conservative management was considered appropriate for our case as the patient had minimal symptoms. She was started on nutritional support (pediasure complete formula and high calorie food as suggested by our nutrition specialist), iron supplements and scheduled for follow up visits every three months to evaluate BP, complete blood count and kidney function tests.

## Discussion and conclusion

Hematuria is a common urinary abnormality in children with a prevalence rate of 0.5–2.0% among school-age children [11]. A good medical history, physical examination and focused laboratory investigations for persistent or recurrent gross hematuria commonly leads to the diagnosis of a long list of causes including infection, glomerulonephritis, stones, trauma, etc. Though the rare causes of gross hematuria maybe missed without a more detailed workup [2, 3].

NCS is a rare cause of hematuria in children. Shin et al. [12] reported isolated hematuria in 33.3% of children with NCS with microhematuria being four times more common than macrohematuria. Its exact incidence and prevalence are difficult to define since the majority of patients are asymptomatic and are diagnosed incidentally on imaging ordered for other reasons [6, 12]. Affected persons belong to different age groups ranging from children up to the seventh decade of life [2].

Nutcracker phenomenon (NCP) or renal vein entrapment syndrome refers to extrinsic narrowing of the LRV between the abdominal aorta and proximal superior mesenteric artery causing outflow disruption from the LRV into the inferior vena cava (IVC) without clinical symptoms [2, 7]. It is not a hereditary phenomenon, although coincidental cases in siblings have been described [7, 13].

The term NCS is used for patients with nutcracker anatomy who present with the characteristic clinical symptoms such as hematuria, proteinuria, flank pain, abdominal pain, and varicocele [1, 7].

The common anterior NCS is created by the entrapment of LRV between AA and SMA. Posterior NCS is rare, resulting from the compression of retro-aortic LRV by abdominal aorta and vertebral column [2, 3, 6–9]. Mallat et al. [6] reported other variations of NCS; anteroposterior NCS and Wilkie syndrome (SMA syndrome) in association with anterior NCS which results from compression of the third portion of the duodenum between the aorta and the SMA [2].

Passage of the renal vein in the acute mesoaortic angle is a normal anatomical finding, but the presence of renal vein compression in few patients is still not clearly understood [3, 13, 14]. Reasons for entrapment include renal ptosis, abnormal division of the left renal vein at a high level, a narrow mesoaortic angle, a pancreatic mass, lymph node enlargement, lack of retroperitoneal adipose tissue and abnormal branching of the SMA from the aorta [2]. LRV hypertension - resulting from outflow blockage to the IVC secondary to LRV entrapment between AA and SMA - is the most commonly pronounced pathogenic mechanism causing varices and collaterals formation in the nearby renal calyces [3, 5, 14]. Hematuria and orthostatic proteinuria with or without flank pain are the consequences of these venous sinuses [7]. Intermittent macroscopic or microscopic hematuria, with occasional need for blood transfusion, is the most commonly reported symptom in NCS [1, 12, 14]. It is attributed to rupture of thin-walled septum between the varices and the collecting system in the renal fornix [7] and may be intermittent in nature due to the relative increase of venous hypertension, which may worsen symptoms after periods of physical exertion or while in an upright position [2]. Orthostatic proteinuria could be related to the increased liberation of angiotensin II and norepinephrine induced by changes in renal hemodynamic upon standing and increased LRV pressure and mild subclinical immune damage [9].

NCS may show atypical presentation as abdominal pain, pelvic or scrotal discomfort due to varicocoele or ovarian/gonadal vein syndrome (Part of pelvic congestion syndrome), dysmenorrhea, fatigue, and orthostatic intolerance [2, 7, 14]. Takemura et al. [4] reported hypotension, syncope, headache, and tachycardia as a rare manifestation of autonomic dysfunction.

NCS associations as Henoch-Schönlein purpura, IgA nephropathy, membranous nephropathy, and idiopathic hypercalciuria with nephrolithiasis have been reported [2, 7].

Children with NCS are usually asymptomatic but others may have severe and persistent symptoms [8, 12]. The severity of the clinical symptoms varies depending on the stages of the pathologic process [2].

NCS is difficult to diagnose due to lack of a diagnostic criteria and patients usually present with non-specific findings creating an overlap with other clinical entities [2]. Sometimes the patient can be referred to various specialty clinics without identifying the cause [2]. Laboratory evaluation is non-diagnostic as well as a single imaging method [1, 2, 14]. Several imaging methods are used to diagnose NCS; Doppler US, CTA, MRA, and retrograde venography [1, 7]. Color Doppler ultrasonography can be used as the first diagnostic test in screening patients with suspected NCS with a sensitivity of 50–78% and specificity of 100% [7, 9, 14].

Despite having a risk of radiation exposure, retrograde venography remains the gold standard for the diagnosis of NCS. It confirms the anatomical changes and measures the pressure gradient across the area of entrapment. It is commonly used in patients with severe symptoms [7]. 3D CTA and MRA are still considered the most reliable non-invasive methods for the diagnosis of NCS [3, 14]. CTA and MRA reveal LRV entrapment and compression showing the “beak sign” which is the abrupt narrowing of the LRV with a triangular shape at the aortomesenteric portion, the development of the collateral vein network, and the abnormal origin of LRV or SMA [1, 3, 7]. Buschi et al. [15] considered a nutcracker effect if the diameter of the LRV lateral to the aorta was more than 50% greater than the diameter of the part crossing in front of the aorta. He showed that the ratio of LRV diameters of the distended to the narrowed portions might reach 4:1. Furthermore, a decreased aortomesenteric angle (AMA) and a shortened aortomesenteric distance can suggest the diagnosis [15]. Mean SMA angle in children varies from different studies. Earlier studies reported that the mean aortomesenteric angle is 38° to 56° [2] while later studies reported it to be 45.8 ± 19.6° [16] and others reported it to be close to 90° in healthy individuals [3]. This variability in AMA can be explained by variability in the patient position during the examination, with greater compression of the LRV by the SMA being in an erect position [2]. In our patient, CTA showed a ratio of 6:1 between the distended and the narrowed portions of LRV and the AMA was 31.4°.

Treatment decision should be based on the severity of symptoms, patient’s age, and the degree of LRV hypertension [4, 6, 7]. Conservative management and close observation is the best option for patients with intermittent hematuria, insignificant flank pain, and normal hemoglobin; especially young patients less than 18-year-old [2, 3, 5]. Complete resolution of hematuria was noticed in 75% of young patients during a period of two years [2]. Several studies have demonstrated that spontaneous resolution of hematuria and proteinuria might be related to increasing body mass index (BMI) during the developmental period [5, 8, 17]. However, another study showed a poor relationship between SMA angle and body fat parameters in children [17]. Etiologic explanations for this phenomenon in developing children relate to a relative paucity of retroperitoneal fat causing ‘bow-stringing’ of the LRV across the aortomesenteric angle due to more posterior position of the left kidney. Subsequent development resulting in increased height and body mass index serves to create a more favorable hemodynamic situation for the LRV and typically lead to symptom resolution [1, 8].

Surgical intervention in NCS including: endovascular stenting, nephrectomy, nephropexy, reno-caval reimplantation or auto-transplantation, transposition of the LRV or SMA, and gonado-caval bypass [3, 6, 14] is indicated in case of recurrent gross hematuria with anemia, severe flank pain, renal functional impairment, and inefficacy or aggravation of symptoms despite conservative treatment such as persistent orthostatic proteinuria after 24 months of follow-up [7].

In conclusion, NCS is a rare cause of recurrent painless macroscopic hematuria in children. It requires a high index of suspicion and knowledge of its various clinical manifestations so as to avoid late diagnosis. Late diagnosis leads to increased financial and psychological burden on the family of the child due to multiple unnecessary investigations and consultations. Imaging studies such as Doppler US and CTA should be performed in such patients to reach an accurate diagnosis with the least invasive procedures.

## Data Availability

Not applicable.

## Abbreviations

AA: Abdominal aorta
AMA: Aortomesenteric angle
BMI: Body mass index
BP: Blood pressure
CRP: C- reactive protein
CTA: Computerized tomography angiography
HPF: High power field
IVC: Inferior vena cava
KFT: Kidney function tests
LRV: Left renal vein
MRA: Magnetic resonance angiography
NCP: Nutcracker phenomenon
NCS: Nutcracker syndrome
SMA: Superior mesenteric artery
UA: urine analysis
US: Ultrasound
